# Region- and voxel-based quantification in human brain of [^18^F]LSN3316612, a radioligand for *O*-GlcNAcase

**DOI:** 10.1186/s13550-021-00780-z

**Published:** 2021-04-01

**Authors:** Jae-Hoon Lee, Mattia Veronese, Jeih-San Liow, Cheryl L. Morse, Jose A. Montero Santamaria, Mohammad B. Haskali, Sami S. Zoghbi, Victor W. Pike, Robert B. Innis, Paolo Zanotti-Fregonara

**Affiliations:** 1 Molecular Imaging Branch, National Institute of Mental Health, National Institutes of Health, 10 Center Drive, Bethesda, MD 20892 USA; 2 Department of Nuclear Medicine, Gangnam Severance Hospital, Yonsei University College of Medicine, Seoul, Republic of Korea; 3 Department of Neuroimaging, Institute of Psychiatry, Psychology, and Neuroscience, King’s College London, London, UK

**Keywords:** *O*-GlcNAcase, Positron emission tomography, Tau, [^18^F]LSN3316612, Parametric image

## Abstract

**Background:**

Previous studies found that the positron emission tomography (PET) radioligand [^18^F]LSN3316612 accurately quantified *O*-GlcNAcase in human brain using a two-tissue compartment model (2TCM). This study sought to assess kinetic model(s) as an alternative to 2TCM for quantifying [^18^F]LSN3316612 binding, particularly in order to generate good-quality parametric images.

**Methods:**

The current study reanalyzed data from a previous study of 10 healthy volunteers who underwent both test and retest PET scans with [^18^F]LSN3316612. Kinetic analysis was performed at the region level with 2TCM using 120-min PET data and arterial input function, which was considered as the gold standard. Quantification was then obtained at both the region and voxel levels using Logan plot, Ichise's multilinear analysis-1 (MA1), standard spectral analysis (SA), and impulse response function at 120 min (IRF_120_). To avoid arterial sampling, a noninvasive relative quantification (standardized uptake value ratio (SUVR)) was also tested using the corpus callosum as a pseudo-reference region. Venous samples were also assessed to see whether they could substitute for arterial ones.

**Results:**

Logan and MA1 generated parametric images of good visual quality and their total distribution volume (*V*_T_) values at both the region and voxel levels were strongly correlated with 2TCM-derived *V*_T_ (*r* = 0.96–0.99) and showed little bias (up to − 8%). SA was more weakly correlated to 2TCM-derived *V*_T_ (*r* = 0.93–0.98) and was more biased (~ 16%). IRF_120_ showed a strong correlation with 2TCM-derived *V*_T_ (*r* = 0.96) but generated noisier parametric images. All techniques were comparable to 2TCM in terms of test–retest variability and reliability except IRF_120_, which gave significantly worse results. Noninvasive SUVR values were not correlated with 2TCM-derived *V*_T_, and arteriovenous equilibrium was never reached.

**Conclusions:**

Compared to SA and IRF, Logan and MA1 are more suitable alternatives to 2TCM for quantifying [^18^F]LSN3316612 and generating good-quality parametric images.

## Introduction

Neurofibrillary tangles—aggregates of hyperphosphorylated tau protein—are a typical feature of Alzheimer's disease (AD) and other tauopathies [1]. The process of *O*-GlcNAcylation [2] involves attaching O-linked β-N-acetylglucosamine *(O*-GlcNAc) at the serine and threonine residue of tau protein [3]. Two enzymes modulate this process: *O*-GlcNAc transferase and *O*-GlcNAc hydrolase (*O*-GlcNAcase, OGA), which catalyze the attachment and detachment of *O*-GlcNAc, respectively. Because this process modifies the key protein involved in tauopathies, and because OGA is located on chromosome 10q24.1 (a locus associated with late-onset AD [4, 5]), interest has grown in *O*-GlcNAcylation as a therapeutic target. Notably, *O*-GlcNAcylation hinders abnormal phosphorylation and aggregation of tau, thus stabilizing the microtubule-associated tau protein [6, 7]. Indeed, tau-specific and overall *O*-GlcNAc are decreased in the brains of individuals with AD [8, 9]. Preclinical studies have also demonstrated that upregulation of *O*-GlcNAcylation by OGA inhibitors reduces pathologic tau phosphorylation and aggregation and prevents neurodegeneration [10–12]. These findings suggest that OGA inhibition may be a potential strategy for treating tauopathies [13, 14].

Our laboratory recently characterized [^18^F]LSN3316612 (fluorine-18-*N*-(5-(((2*S*,4*S*)-2-methyl-4-(6-fluoropyridin-2-yloxy) piperidin-1-yl)methyl)thiazol-2-yl)acetamide), a positron emission tomography (PET) radioligand capable of measuring OGA in brain. An initial in vitro study demonstrated that [^18^F]LSN3316612 was a highly specific and potent OGA ligand [15] with good imaging properties in nonhuman primates [16]. Human studies subsequently confirmed that [^18^F]LSN3316612 was an excellent PET radioligand for imaging OGA in vivo. Specifically, brain uptake was well quantified as total distribution volume (*V*_T_) using the two-tissue compartment model (2TCM); *V*_T_ measures showed low variability and moderate reliability under test and retest conditions, and *V*_T_ was stable about two hours after injection, suggesting no significant radiometabolite accumulation in brain [17]. However, only *V*_T_ derived from compartment modeling was examined at the region level, and 2TCM is not suitable for voxel-wise analysis due to its high computational demand [18]. Thus, the feasibility of other kinetic models remains unknown, particularly with regard to generating high-quality parametric images of [^18^F]LSN3316612 binding. The present study used the previously obtained test–retest dataset [17] to identify which kinetic model(s) yield binding parameters that are close to 2TCM-derived *V*_T,_ which is considered the gold standard for quantifying [^18^F]LSN3316612 binding.

## Materials and methods

### Participants

The current study reanalyzed data from 10 healthy volunteers (five males, five females; 43 ± 11 years) who had enrolled in a previous protocol (NCT03632226) [17] and underwent both test and retest PET scans with [^18^F]LSN3316612. The National Institutes of Health (NIH) Combined Neurosciences Institutional Review Board approved the original study (Protocol 16-M-0105), and informed consent was obtained from all participants.

### Imaging studies

Details regarding the participants, PET study protocol and processing, and measurement of [^18^F]LSN3316612 in plasma are described in detail in the previously published paper [17]. Briefly, all participants underwent dynamic PET scans using an mCT scanner (Siemens Medical Solutions, Cary, NC, USA) after intravenous administration of [^18^F]LSN3316612. Data were reconstructed into 45 frames (6 × 0.5 min, 3 × 1 min, 2 × 2 min, 34 × 5 min) using a three-dimensional ordered subset expectation maximization algorithm. For the retest study, participants were scanned on a separate day (median interval = 49 ± 48 days (range 8–150)).

For structural magnetic resonance (MR) imaging, sagittal T1-weighted brain MR images were obtained in all participants using a 3 T Philips Achieva scanner (Philips Healthcare; Andover, MA) with turbo field echo sequence (repetition time = 8.1 ms, echo time = 3.7 ms, flip angle = 8, matrix = 181 × 256 × 256, voxel size = 1.000 × 0.983 × 0.983 mm).

Image preprocessing—such as motion correction, MR segmentation, coregistration between PET and MR, and atlas normalization—was performed using the PNEURO pipeline implemented in PMOD 3.903 (PMOD, Zurich, Switzerland). A total of 83 predefined regions-of-interest (ROIs) from the Hammers N30R83 maximum probability atlas [19] were adjusted to the MRIs of individual participants and subsequently combined into an individual template comprising 16 consolidated regions. For region-level analyses, brain time-activity curves were obtained by applying the individual ROI template on the dynamic PET images transformed into MR space. For voxel-level analyses, parametric images were obtained with the PXMOD tool, and the average value over each brain region was calculated by overlaying the same Hammers atlas used for the region-level analyses onto the parametric images.

Arterial blood was concomitantly withdrawn during the brain PET scan. Whole and plasma concentrations of [^18^F]LSN3316612 were measured using an automatic gamma counter and corrected after radiometabolites were separated using high-performance liquid chromatography (HPLC). Both whole-blood and total plasma activity curves were then fitted to a tri-exponential function, and the plasma parent fraction was fitted using a Hill function [20]. Parent radioactivity in plasma, which was implicitly generated by the product of the total plasma activity curve and parent fraction in PMOD, was used as the input function.

### Kinetic models

The acquisition lasted for 180 min; however, only the first 120 min of PET and blood data were used for kinetic evaluation because our previous study had demonstrated that 120 min of brain and plasma data were sufficient to measure the *V*_T_ of [^11^C]LSN3316612 with small bias and good identifiability [17].

#### Requiring blood input function

Because the 2TCM is superior to the one-tissue compartment model for quantifying [^18^F]LSN3316612 binding [17], the gold standard for the present study was total *V*_T_ calculated with 2TCM. Brain activity was corrected for its vascular components, assuming that blood volume was 5% of total brain volume [21]. The delay in radioligand arrival between the radial artery and the brain was corrected by fitting the whole-brain time-activity curve.

Regional *V*_T_ values were also obtained with the Logan plot (Logan_voi_), Ichise's multilinear analysis-1 (MA1_voi_), and spectral analysis (SA_voi_). In addition to *V*_T_, SA was used to calculate the impulse response function at 120 min (IRF_120_) (see Appendix).

Similar to region-level analyses, voxel-wise parametric images were created using the following four methods: Logan plot (Logan_voxel_), MA1 (MA1_voxel_), SA (SA_voxel_), and IRF (IRF_120voxel_). For Logan_voxel_ and MA1_voxel_, starting PET frames used for regression were selected based on t* of the whole brain. Regional binding parameters—*V*_T_ and IRF_120_—were then obtained by measuring the average value of the voxels in each region.

#### No requirement for input function

For relative measurement of [^18^F]LSN3316612 binding, time-averaged PET images at 20-min intervals were created starting from 60 min (60–80, 80–100, and 100–120 min). Standardized uptake value (SUV) was measured for each ROI, and regional SUV ratio (SUVR) was then calculated by dividing the SUV of each region by that of the corpus callosum. Because OGA is known to be ubiquitously expressed throughout the human brain, a suitable reference region devoid of specific binding does not exist. Therefore, the corpus callosum, which has the lowest *V*_T_, was designated as a normative region for SUV. Among the three time intervals (60–80, 80–100, and 100–120 min), the 100–120-min data showed the best correlation between SUVR and 2TCM-derived *V*_T_ values.

### Assessing the arteriovenous equilibrium of [^18^F]LSN3316612

Venous blood samples were simultaneously obtained in four participants, with arterial sampling at 30, 60, 120, and 180 min. Plasma parent concentration of [^18^F]LSN3316612 was obtained for each of the four timepoints and compared with those obtained from the arterial blood samples. Whether arterial blood samples could be replaced by venous blood samples was explored based on the agreement of the plasma parent concentrations at those four timepoints.

### Statistical analysis

For all participants, the *V*_T_ value obtained with 2TCM at the region level was considered as the gold standard [17]. Pearson’s coefficient (*r*) and percentage bias (calculated as the mean relative difference from 2TCM estimates) were used to assess the correlation and difference between each binding parameter and *V*_T_ calculated from 2TCM, respectively. For the voxel-level analysis, the mean value of each brain region obtained from the parametric images was compared to the corresponding 2TCM-derived *V*_T_ using the same brain atlas.

*V*_T_ comparison among different kinetic models at both region and voxel levels was performed by repeated measures analysis of variance (ANOVA) followed by a Fisher’s least significant difference (LSD) test.

Test–retest variability and absolute test–retest variability were calculated as follows:

$${\text{Test-retest}}\,{\text{variability}}\,(\% ) = \frac{{{\text{Retest}}\,{\text{value}} - {\text{Test}}\,{\text{value}}}}{{\left( {{\text{Retest}}\,{\text{value}} + {\text{Test}}\,{\text{value}}} \right)/2}} \times 100$$

$${\text{Absolute}}\,{\text{test-retest}}\,{\text{variability}}\,(\% ) = \frac{{\left| {{\text{Retest}}\,{\text{value}} - {\text{Test}}\,{\text{value}}} \right|}}{{\left( {{\text{Retest}}\,{\text{value}} + {\text{Test}}\,{\text{value}}} \right)/2}} \times 100$$

To assess test–retest reliability, the intra-class correlation coefficient (ICC) of each region was calculated as follows [22]:

$${\text{ICC}} = \frac{{{\text{BSMSS}} - {\text{WSMSS}}}}{{{\text{BSMSS}} + {\text{WSMSS}}}}$$

where BSMSS and WSMSS are the mean sums of squares between subjects and within subjects, respectively. ICC values between 0.50 and 0.75 were defined as moderate, between 0.75 and 0.90 as good, and above 0.9 as excellent [23].

Statistical significance was set at *P* < 0.05, and all statistical analyses were conducted using GraphPad Prism 5 (GraphPad Software, La Jolla, CA, USA) and SPSS 25 (IBM Corp., New York, USA).

## Results

### Kinetic model comparison

At the region level, the whole-brain *V*_T_ obtained with Logan_voi_, MA1_voi_, and IRF_120voi_ were strongly correlated with *V*_T_ obtained with 2TCM (*r* > 0.95, *P* < 0.001 for all) (Table 1). SA_voi_ showed a significant but slightly weaker correlation (*r* = 0.90, *P* < 0.001).

**Table 1:** Correlation (Pearson's coefficient, *r*) of different binding parameters with *V*_T_ values calculated from the two-tissue compartment model in the representative regions

Model	Pseudo-reference	Region level				Voxel level			
		Logan	MA1	SA	IRF	Logan	MA1	SA	IRF
Parameter	SUVR	*V*_T_	*V*_T_	*V*_T_	IRF_120_	*V*_T_	*V*_T_	*V*_T_	IRF_120_
Whole brain	− 0.244^ns^	0.979	0.986	0.903	0.960	0.983	0.955	0.980	0.955
Temporal	− 0.236^ns^	0.965	0.979	0.922	0.970	0.979	0.923	0.990	0.970
Frontal	− 0.261^ns^	0.976	0.986	0.889*	0.967	0.982	0.938	0.958	0.957
Cerebellum	0.163^ns^	0.983	0.993	0.939	0.942	0.985	0.958	0.989	0.944

*ns*, not significant; *, *P* < 0.01; otherwise, *P* < 0.001

SUVR, ratio of standardized uptake value (SUV) of each region measured from the time-averaged PET images of 100 to 120 min to that of the pseudo-reference region (the corpus callosum); MA1, Ichise’s multilinear analysis-1; SA, standard spectral analysis; *V*_T_, total distribution volume (mL∙cm^−3^); IRF_120_, impulse response function (IRF) calculated at 120 min. Regional *V*_T_ estimates from parametric mapping methods were calculated as the mean of *V*_T_ voxel estimates in the region

In contrast, SUVR calculated using a pseudo-reference region did not correlate with 2TCM-derived *V*_T_ (*r* = − 0.24, *P* = 0.497) and was thus excluded from further analyses. A pseudo-reference region is a region that has specific binding but is relatively unaffected by the disease [24, 25]. Logan_voi_ and MA1_voi_ slightly underestimated *V*_T_ values; the bias relative to *V*_T_ obtained with 2TCM was − 4.8% (95% confidence interval (CI), − 5.5 to − 4.1%; range − 3.0 to − 8.3%) for Logan_voi_ and − 8.3% (95% CI − 9.3 to − 7.4%; range − 6.2 to − 13.5%) for MA1_voi_ (Table 2). In contrast, SA_voi_ overestimated *V*_T_ with a bias of 11.6% (95% CI, 10.8 to 12.5%; range 7.7 to 13.9%). However, repeated measures ANOVA determined that the differences in *V*_T_ were not statistically significant among 2TCM, Logan_voi_, MA1_voi_, and SA_voi_ (*F*(3, 36) = 2.340, *P* = 0.090).

**Table 2:** Comparison of the regional *V*_T_ values obtained with different kinetic methods at region and voxel levels

	2TCM	Region level			Voxel level		
		Logan	MA1	SA	Logan	MA1	SA
Whole brain	13.2 ± 2.4	12.7 ± 2.4 (− 4.8)	12.2 ± 2.2 (− 8.3)	14.9 ± 2.7 (11.6)	12.3 ± 2.0 (− 7.3)	13.0 ± 2.6 (− 2.4)	15.4 ± 2.7 (15.5)
Temporal	15.0 ± 2.8	14.2 ± 2.9 (− 5.6)	13.6 ± 2.6 (− 9.7)	16.8 ± 2.6 (12.4)	13.6 ± 2.4 (− 9.4)	14.7 ± 3.3 (− 2.5)	17.4 ± 2.9 (15.6)
Frontal	14.1 ± 2.5	13.5 ± 2.6 (− 4.8)	13.0 ± 2.3 (− 8.3)	15.9 ± 3.0 (11.8)	13 ± 2.2 (− 8.0)	13.7 ± 2.9 (− 3.4)	16.4 ± 3.0 (15.0)
Striatum	15.6 ± 3.2	14.7 ± 3.2 (− 5.8)	14.2 ± 3.0 (− 9.2)	17.8 ± 3.4 (13.9)	14 ± 2.5 (− 10.2)	14.7 ± 3.1 (− 5.8)	17.6 ± 3.3 (12.8)
Cerebellum	14.1 ± 2.2	13.4 ± 2.3 (− 5.1)	12.9 ± 2.0 (− 8.9)	15.4 ± 2.3 (9.3)	12.9 ± 2.1 (− 9.1)	13.5 ± 2.7 (− 4.7)	16.1 ± 2.7 (13.0)
Brainstem	12.2 ± 2.3	11.7 ± 2.3 (− 4.1)	11.3 ± 2.1 (− 7.3)	13.2 ± 2.7 (7.7)	11.5 ± 2.0 (− 5.6)	12.0 ± 2.4 (− 1.5)	14.0 ± 2.5 (14.5)

*V*_T_ is presented as a mean ± SD (mL∙cm^−3^) and a bias (%) in parentheses; bias was calculated as the percentage ratio of the difference in *V*_T_ between 2TCM and each quantification method to their average. *V*_T_, total distribution volume; 2TCM, two-tissue compartment model; MA1, Ichise’s multilinear analysis-1; SA, standard spectral analysis

**Fig. 1:**
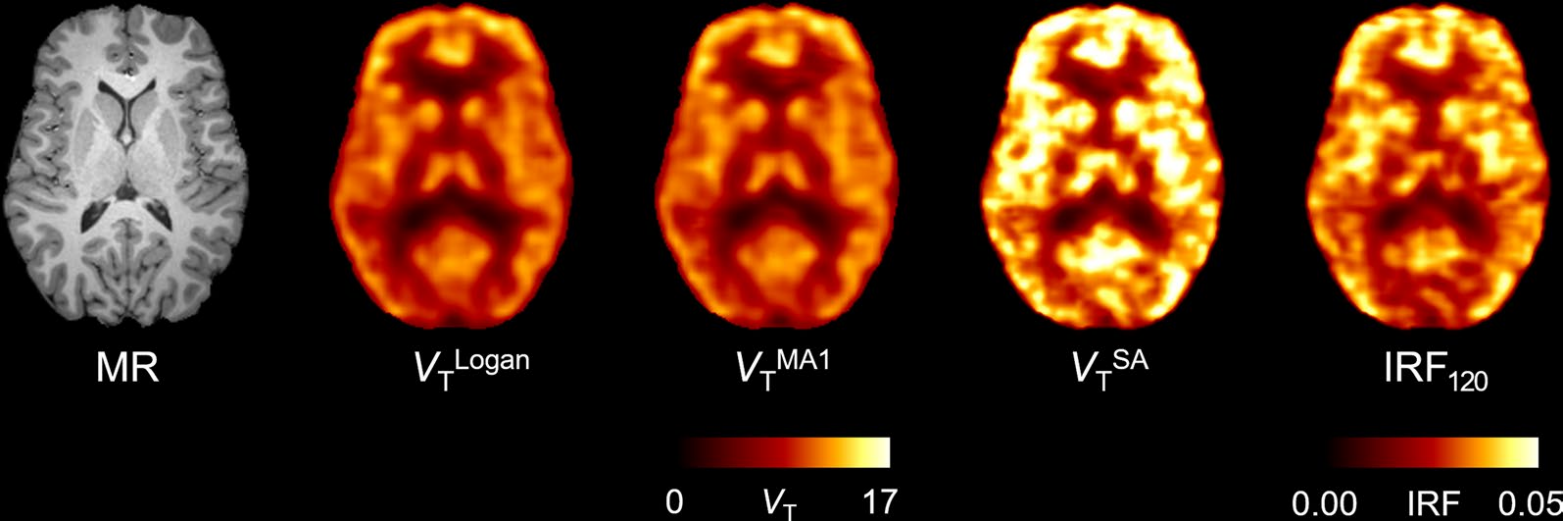
The trans-axial parametric images generated by different kinetic models in a single healthy volunteer. Total distribution volume (*V*_T,_ mL∙cm^−3^) was obtained with Logan (*V*_T_^Logan^), multilinear analysis-1 (*V*_T_^MA1^), and standard spectral analysis (*V*_T_^SA^_)_. Impulse response function was calculated at 120 min (IRF_120_)

At the voxel level, Logan, MA1, and SA generated parametric images of good quality that represented OGA density in the brain (Fig. 1). The parametric images generated by IRF_120voxel_ were noisier, although the overall image quality was acceptable for quantification. Whole-brain *V*_T_ values obtained with Logan_voxel_, MA1_voxel_, SA_voxel_, and IRF_120_ were strongly correlated with *V*_T_ obtained with 2TCM (*r* > 0.95, *P* < 0.001 for all; Table 1). Logan_voxel_ and MA1_voxel_ showed a small bias in *V*_T_ with 2TCM: − 7.3% (95% CI − 9.2 to − 5.5%; range − 5.6 to − 20.4%) for Logan_voxel_ and − 2.4% (95% CI − 3.6 to − 1.2%; range − 7.3% to 1.6%) for MA1_voxel_ (Table 2). SA_voxel_ overestimated *V*_T_ with a bias of 15.5% (95% CI, 14.6 to 16.4%; range 9.3 to 17.3%). The repeated measures ANOVA was significant (*F*(3, 36) = 3.206, *P* = 0.034) and Fisher’s LSD test showed that SA differed from both Logan and MA1. Indeed, SA tended to overestimate *V*_T_, whereas graphical analyses underestimate *V*_T_ compared to compartmental modeling.

Although both test and retest datasets were analyzed separately in the same way, only the test data are reported here because the retest results were very similar (data not shown).

### Test–retest variability and reliability

*V*_T_ obtained with Logan, MA1, and SA showed low test–retest variability and moderate test–retest reliability, similar to those obtained with 2TCM-derived *V*_T_. At the region level, the absolute test–retest variability ranged from 10.7 to 11.5%, and ICC ranged from 0.69 to 0.73, compared to 11.7% and 0.70 for 2TCM, respectively (Table 3). At the voxel level, the absolute test–retest variability ranged from 10.4% to 12.0%, and ICC ranged from 0.58 to 0.80; these values demonstrate moderate to good reliability. In contrast, IRF_120_ showed significantly worse test–retest variability (19.7% to 20.8%) than the other techniques at both the region and voxel levels, though it had comparable ICC (0.71).

**Table 3:** Test–retest variability and reliability of different parameters of [^18^F]LSN3316612 binding

	Test^a^	Retest^a^	TRV (%)	aTRV (%)	ICC
Region level					
*V*_T_^2TCM^	13.2 (17.8)	14.7 (20.1)	10.1	11.7	0.70
*V*_T_^Logan^	12.7 (19.0)	14.0 (19.6)	10.1	10.7	0.73
*V*_T_^MA1^	12.2 (17.8)	13.5 (19.7)	10.2	11.2	0.69
*V*_T_^SA^	14.9 (18.3)	16.7 (16.2)	11.5	11.5	0.71
IRF_120_	0.04 (32.4)	0.04 (25.3)	16.4	20.8	0.71
Voxel level					
*V*_T_^Logan^	12.3 (16.4)	13.7 (20.0)	10.1	12.0	0.58
*V*_T_^MA1^	13.0 (20.4)	14.1 (20.5)	8.4	10.5	0.80
*V*_T_^SA^	15.4 (17.2)	17.0 (15.2)	10.2	10.4	0.74
IRF_120_	0.04 (32.2)	0.04 (24.7)	15.8	19.7	0.71

TRV, test–retest variability; aTRV, absolute test–retest variability; ICC, intraclass correlation coefficient; *V*_T_, total distribution volume; 2TCM, two-tissue compartment model; MA1, multilinear analysis-1; SA, standard spectral analysis; IRF_120_, impulse response function calculated at 120 min

^a^Data are presented as *V*_T_ (mL∙cm^−3^) with a coefficient of variation (%) in parentheses

### Use of venous input function

**Fig. 2:**
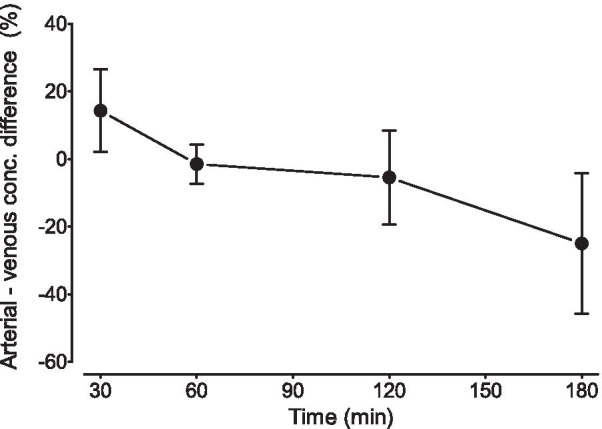
The arterial-venous difference in radioligand radioactivity concentrations (mean ± SD) in four healthy volunteers

Equilibrium was not reached between arterial and venous blood data measured at four timepoints. At 30 min, arterial blood samples showed higher plasma concentrations than venous blood samples, with an average arterial-venous difference of 14.3 ± 12.2% (range 7.3 to 32.5%). Plasma concentrations were almost equal at 60 min, with an average difference of − 1.5 ± 5.9% (range − 8.0 to 0.0%) between arterial and venous blood data; however, at later timepoints, the average difference became greater (− 5.5 ± 14.0% (range − 13.5 to 15.4%) at 120 min and − 25.0 ± 21.0% (range − 54.7 to − 6.7%) at 180 min (Fig. 2).

## Discussion

The present study—which used previous data to explore different kinetic model(s) for quantifying [^18^F]LSN3316612 binding—found that Logan and MA1 were the models whose *V*_T_ estimations were the closest to those obtained using the gold standard of 2TCM, particularly with regard to generating good-quality parametric images. Compared to SA, Logan and MA1 showed the smallest bias of *V*_T_ compared to 2TCM. Although *V*_T_ values from all techniques were strongly correlated with 2TCM-derived *V*_T_ at both the region and voxel levels, the biases for Logan and MA1 were generally within − 8% of the 2TCM-derived *V*_T_ values, while SA showed a larger bias (~ 16%). In contrast, while IRF_120_ was strongly correlated with 2TCM-derived *V*_T_ in both region- and voxel-wise analyses, its test–retest variability was significantly higher than for the other techniques. Therefore, IRF_120_ should not be considered a viable alternative for [^18^F]LSN3316612 quantification. Finally, the lack of correlation between SUVR and 2TCM-derived *V*_T_ values and the absence of arteriovenous equilibrium 180 min post-injection suggest that arterial sampling is needed to quantify [^18^F]LSN3316612 scans.

Previous studies from our laboratory showed that [^18^F]LSN3316612 is suitable for clinical research [17]. Specifically, brain uptake was high and could be well-quantified as *V*_T_ with 2TCM. Furthermore, *V*_T_ was stable over time and showed low variability and fair reliability under test–retest conditions. However, quantifying increased OGA density during disease development and progression requires not only favorable kinetic properties but also validation of an appropriate analytic methodology. In this context, voxel-based analyses may enable early detection compared to region-based analyses. Theoretically, region-based analyses may be appropriate if a study has an a priori hypothesis about a specific brain region. However, voxel-based analyses may identify changes in smaller and isolated areas that take place during early disease stages. Indeed, a change in a subset of voxels may be smoothed out when all voxel values are averaged together to a large ROI. Moreover, voxel-based analyses examine the whole brain without a priori spatial assumption and are more appropriate for explorative studies of radioligands that bind across all brain regions, such as [^18^F]LSN3316612. Voxel-based analyses are more affected by noise, and adjacent voxels are not statistically independent observations because they are spatially correlated due to the finite resolution of the scanner. In our study, however, the impact of noise at the voxel level was minimized by merging the individual voxels of the parametric images into relatively noiseless large regions.

Although 2TCM is considered to be the gold standard for quantifying [^18^F]LSN3316612 in the brain, it is unsuitable for voxel-level analyses. The conventional nonlinear least-squares fitting is susceptible to the high noise encountered at the voxel level [26]. As an example, Rizzo and colleagues [27] found that generating parametric images of [^11^C](*R*)-rolipram with 2TCM took about 20 h of computing time per participant and resulted in a map where more than half of voxels had to be discarded because of outlying values. Parametric images may also be generated using the basis function derived from 2TCM [28], but the results may be inaccurate and are still computationally challenging [18]. Alternative methods were recently proposed for computing all rate constants of 2TCM within a reasonable time and improving robustness to measurement noise, but they require validation in other reversible radioligands [18, 29].

In this context, the present study found that Logan and MA1 were valid alternatives to 2TCM at the region level and suitable for generating parametric images. Logan and MA1 use a linear estimator, thus enabling the rapid estimation of macro-parameters, such as *V*_T_ [30, 31]. Moreover, these graphical approaches do not require a priori knowledge of compartmental configurations. One drawback of the graphical analyses is that they underestimate *V*_T_ due to noise in the PET data, especially for Logan [32]. In our study, however, the bias was both small (up to about 8%) and similar between region-based and voxel-based analyses.

Interestingly, SA similarly uses a linear estimator and does not depend on specific compartment model configuration, thus making it both computationally rapid and widely applicable to PET radioligand research. However, in the present study, SA overestimated *V*_T_ by about 16%. SA is well known to be sensitive to noise in the data [33], with a tendency to overestimate macro-parameters like *V*_T_, especially for shorter acquisition times.

Reliable quantification of [^18^F]LSN3316612 requires arterial blood sampling. To eliminate the need for this invasive and burdensome procedure, the present study examined use of a pseudo-reference region and also measured simultaneous arterial and venous concentrations in four participants. Because pseudo-reference regions are relatively unaffected by the disease [32, 33], this approach can only be properly validated using groups of both patients and healthy controls [34]. However, given the very poor correlation with *V*_T_ already present in healthy participants, a pseudo-reference region may not be a viable option for [^18^F]LSN3316612. Not only was a suitable arteriovenous equilibrium never reached over 180 min, but considerable inter-subject variability was also observed. However, this lack of suitable equilibrium between the artery and vein is common for radioligands [35]. Even if an average equilibrium is obtained at a late timepoint, considerable inter-subject variability will result in *V*_T_ estimation errors in one or more participants. Given the often limited number of participants in PET studies, the presence of these outliers could easily compromise results.

Finally, and as previously reported [17], *V*_T_ was generally higher in the second scan compared to the first, regardless of the method used. If this were purely due to chance, the probability of obtaining nine higher retest scans from 10 subjects would be smaller than 1%. Curiously, Coughlin and colleagues similarly found a systematic higher *V*_T_ value in retest scans compared to test scans when scanning six subjects with the TSPO radioligand [^11^C]DPA-713 [36]. In their study—and in contrast to ours—all retest scans were acquired on the same day as the test scan. The authors speculated that these findings might be due to factors such as hormone-mediated phasic changes in TSPO expression, tonic changes due to stress/anxiety related to the procedure, or alterations in blood cholesterol levels due to food intake between the scans. As yet unidentified physiological variables may also explain our results.

## Conclusions

Logan plot and MA1 showed the smallest *V*_T_ bias when compared with 2TCM at the region and voxel levels and generated good-quality parametric images. Therefore, both models were better than SA or IRF for quantifying [^18^F]LSN3316612 uptake in human brain.

## Data Availability

The data that support the findings of this study will be made available, without restriction, by request to Dr. Robert Innis (innisr@mail.nih.gov). The data are not publicly available due to privacy and ethical restrictions.

## Appendix

*Logan plot.* The Logan plot is a model-independent graphical method for reversible tracers [30]. It performs a linearization of the data so that, after a certain time (*t* > *t**), the slope can be related to the *V*_T_, according to the following equation:

$$\begin{array}{*{20}c} {\frac{{\mathop \smallint \nolimits_{0}^{t} C_{T} \left( \tau \right){\text{d}}\tau }}{{C_{T} \left( t \right)}} = V_{T} \times \frac{{\mathop \smallint \nolimits_{0}^{t} C_{P} \left( \tau \right){\text{d}}\tau }}{{C_{T} \left( t \right)}} + b} \\ \end{array}$$

where *C*_*T*_*(t)* is the tissue time-activity curve, *C*_*P*_*(t)* is the metabolite-corrected arterial input function, the slope (*V*_*T*_) is the total distribution volume, and *b* is the intercept, which becomes constant for *t* > *t**.

*Ichise's multilinear analysis.* The multilinear analysis (MA1) is a modification of the Logan plot aimed at minimizing the bias induced by noise in the measurements [31]:

$$C_{T} \left( t \right) = - \frac{{V_{T} }}{b}\mathop \smallint \limits_{0}^{t} C_{P} \left( \tau \right){\text{d}}\tau + \frac{1}{b}\mathop \smallint \limits_{0}^{t} C_{T} \left( \tau \right){\text{d}}\tau$$

where *C*_*T*_*(t)* represents the tissue time-activity curve, *C*_*P*_*(t)* the metabolite-corrected arterial input function, *V*_*T*_ the total distribution volume, and *b* the intercept of the Logan plot that becomes constant after an equilibration time (*t* > *t**).

*Spectral analysis.* This technique is based on a single time-invariant input/single output model used to identify tissue kinetic components [37]. SA does not require prior knowledge of the number of compartments in the system. In SA, IRF represents the hypothetical concentration of the radioligand that would be measured after ideal instantaneous bolus injection. The tissue concentration, *C*_*T*_*(t)*, is modeled as a convolution of the radiometabolite-corrected arterial input function, *C*_*P*_*(t)*, with the sum of *M* + *1* distinct decreasing exponential function [37]:

$${\text{IRF}}\left( t \right) = \begin{array}{*{20}c} {\mathop \sum \limits_{j = 0}^{M} \alpha_{j} e^{{ - \beta_{j} t}} } \\ \end{array}$$

$$C_{T} \left( t \right) = \mathop \sum \limits_{j = 0}^{M} C_{p} \left( t \right)*\alpha_{j} e^{{ - \beta_{j} t}} = \mathop \sum \limits_{j = 0}^{M} \alpha_{j} \mathop \smallint \limits_{0}^{t} C_{p} \left( t \right)e^{{ - \beta_{j} \left( {t - \tau } \right)}} {\text{d}}\tau$$

where *α*_*j*_ and *β*_*j*_ are functions of the transfer rate constants assumed to be real-valued and nonnegative.

*V*_T_ was calculated from the estimated spectrum as follows:

$$V_{T} = \mathop \sum \limits_{j = 1}^{M} \frac{{\alpha_{j} }}{{\beta_{j} }}$$

In the present study, the *β*_*j*_ grid was defined with a maximum value of 250 and a logarithmic distribution of *β*_*j*_, *j* = 1, 2… *M*, using a spectral range from 0.005 to 5 min^−1^.

In addition, impulse response function at 120 min (IRF_120_) was used as a proxy of the binding of [^18^F]LSN3316612 [38, 39]. SA was performed by using the source codes of SAKE on MATLAB R2016b (MathWorks, Natick, MA, USA) [37].

## Abbreviations

1TCM: One-tissue compartment model
2TCM: Two-tissue compartment model
ANOVA: Analysis of variance
ICC: Intra-class correlation coefficient
IRF_120_: Impulse response function calculated at 120 min
MA1: Ichise's multilinear analysis-1
MR: Magnetic resonance
*O*-GlcNAc: *O*-Linked β-*N*-acetylglucosamine
OGA: *O*-GlcNAc hydrolase (*O*-GlcNAcase)
PET: Positron emission tomography
ROI: Region of interest
SA: Standard spectral analysis
SUV: Standardized uptake value
SUVR: Standardized uptake ratio between the region/voxel and pseudo-reference region
TRV: Test–retest variability
*V*_T_: Total distribution volume
